# Supplementary material for: Intraspecific variation in the diet of the Mexican garter snake Thamnophis eques
Source: PeerJ. 2017 Nov 14;5:e4036. doi: 10.7717/peerj.4036 (PMC5691781; doi:10.7717/peerj.4036)
Supplement: Table S1 [file peerj-05-4036-s002.docx]

Table S1. Collection dates of Mexican Garter Snake during the active reproductive season (February to November) from different years (1980-1986, 1994-1995) at 23 location in Mexican Plateau.

| **Year** | **Month** | **Day** |
| --- | --- | --- |
| 1980 | september | 29 |
| 1981 | april | 3, 19 |
|  | june | 14-15, 24 |
|  | august | 13-14 |
|  | september | 30 |
|  | october | 1, 8-9 |
|  | november | 5-6 |
| 1982 | febrero | 17-18 |
|  | march | 18-20 |
|  | april | 28-30 |
|  | may | 25-26 |
|  | june | 30 |
|  | julio | 1 |
|  | august | 17-18 |
|  | september | 9-11 |
|  | november | 19-20 |
| 1983 | february | 21-22 |
|  | march | 16-17 |
|  | april | 11-12 |
|  | may | 8-9 |
|  | june | 14-15 |
|  | july | 4-6 |
|  | august | 8-10 |
|  | september | 5-6 |
|  | october | 5-6 |
|  | november | 10-11 |
| 1984 | april | 26 |
|  | may | 15 |
|  | june | 27-28 |
| 1985 | april | 4 |
|  | may | 6, 7, 31 |
|  | june | 2, 7 |
|  | august | 5, 27 |
| 1986 | april | 12, 14 |
|  | may | 1 |
| 1994 | january | 21 |
|  | march | 26 |
|  | april | 29 |
|  | may | 27 |
|  | june | 29 |
|  | october | 1 |
| 1995 | july | 15 |
|  | august | 19 |
|  | september | 2-3 |
