# Supplementary material for: Intraspecific variation in the diet of the Mexican garter snake Thamnophis eques
Source: PeerJ. 2017 Nov 14;5:e4036. doi: 10.7717/peerj.4036 (PMC5691781; doi:10.7717/peerj.4036)
Supplement: Table S2 [file peerj-05-4036-s003.docx]

Table S2. Localities on the Mexican plateau from which *T. eques* were collected.

|  | Locality (State) | Geographic Coordinates | | Altitude (m) | |  |
| --- | --- | --- | --- | --- | --- | --- |
| Lerma drainage | | |  | |  | |
| 1 | San Miguel Almaya (State of Mexico) | | 19°12' N, 99°26' W | | 2,688 | |
| 2 | San Pedro Tlaltizapan (State of Mexico) | | 19°11' N, 99°31' W | | 2,575 | |
| 3 | Citlali (State of Mexico) | | 19°27' N, 99°46' W | | 2,560 | |
| 4 | Ignacio Ramirez (State of Mexico) | | 19°27' N, 99°47' W | | 2,550 | |
| 5 | San Antonio Los Remedios (State of Mexico) | | 19°37' N, 99°50' W | | 2,532 | |
| 6 | Pathe (1 km S Acambay) (State of Mexico) | | 19°55' N, 99°49' W | | 2,721 | |
| 7 | Pond 1 Km W Acambay (State of Mexico) | | 19°57' N, 99°52' W | | 2,513 | |
| 8 | Solis (State of Mexico) | | 19°58' N, 100°04' W | | 2,380 | |
| 9 | Chapala, San Pedro Tesistan (Jalisco) | | 20°13' N, 103°24' W | | 1,517 | |
| 10 | Chapala, Isla Petetan (Michoacán) | | 20°09' N, 102°51' W | | 1,520 | |
| 11 | Cuitzeo (Michoacán) | | 19°58' N, 101°03' W | | 1,820 | |
| 12 | Laguna Victoria (State of Mexico) | | 19°15' N, 99°28' W | | 2,584 | |
| 13 | El Oro (State of Mexico) | | 19°49' N, 100°02' W | | 2,567 | |
| 14 | Presa Mortero (State of Mexico) | | 19°48' N, 100°06' W | | 2,672 | |
| 15 | Santa Rosa de Solis (State of Mexico) | | 20°01' N, 100°04' W | | 2,395 | |
| 16 | Laguna Sirahuen (Michoacán) | | 19°26' N, 101°43' W | | 2,095 | |
| Tula drainage | | |  | |  | |
| 17 | San Francisco (State of Mexico) | | 20°02' N, 99°33' W | | 2,434 | |
| 18 | San Andres Timilpan, 2 (State of Mexico) | | 19°53' N, 99°43' W | | 2,633 | |
| 19 | Presa Nopala Shore NE (Hidalgo) | | 20°15' N, 99°39' W | | 2,340 | |
| 20 | Rio Tula at Tezontepec (Hidalgo) | | 20°12' N, 99°16' W | | 1,990 | |
| 21 | Highway Mexico-Queretaro km 86 (Hidalgo) | | 19°58' N, 99°27' W | | 2,340 | |
| 22 | Fuentezuela-Tequisquiapan (Querétaro) | | 20°33' N, 99°54' W | | 1,908 | |
| Nazas drainage | | |  | |  | |
| 23 | Santa Cruz (Zacatecas) | | 23°09' N, 103°01' W | | 2,130 | |
